# Special Issue: Rare Respiratory Diseases: A Personal and Public Health Issue

**DOI:** 10.3390/jcm10245906

**Published:** 2021-12-16

**Authors:** María Magallón, Lucía Bañuls, Silvia Castillo, María Mercedes Navarro-García, Cruz González, Francisco Dasí

**Affiliations:** 1 Research Group on Rare Respiratory Diseases (ERR), Instituto de Investigación Sanitaria INCLIVA, Fundación Investigación Hospital Clínico Valencia, Avda. Menéndez y Pelayo, 4, 46010 Valencia, Spain; mariamagallon94@gmail.com (M.M.); lucia.banyuls.soto@gmail.com (L.B.); sccorullon@gmail.com (S.C.); mer_navarro2002@yahoo.es (M.M.N.-G.); Cruz.Gonzalez@uv.es (C.G.); 2 Research Group on Rare Respiratory Diseases (ERR), Department of Physiology, School of Medicine, University of Valencia, Avda. Blasco Ibáñez, 15, 46010 Valencia, Spain; 3 Paediatrics Unit, Hospital Clínico Universitario de Valencia, Avda. Blasco Ibáñez, 17, 46010 Valencia, Spain; 4 Pneumology Unit, Hospital Clínico Universitario de Valencia, Avda. Blasco Ibáñez, 17, 46010 Valencia, Spain

In the 1970s, the term “rare disease” was coined to describe a category of inherited metabolic diseases with low prevalence and a wide range of symptoms. The majority of rare diseases are life-threatening and have a considerable impact on a patient’s quality of life. Many of them are fatal. Although rare diseases are uncommon, they affect millions of people worldwide, and the available information on them is often insufficient, consisting of a few isolated clinical cases. The term “low prevalence” is defined differently in different countries. A disease is considered rare in the European Union when the prevalence is fewer than 5 cases per 10,000 people. In the United States, the Orphan Drug Act defines a rare disease as one that affects fewer than 200,000 people. Other countries, such as Japan, prefer to employ a stricter threshold, such as fewer than 4 cases per 10,000 people. The number of rare diseases is estimated to be between 5000 and 8000, with the majority of them being genetic and having a hereditary component, while some occur due to exposure to infectious agents, toxins, or severe treatment side-effects. Between 3.5% and 5.9% of the world’s population is affected by rare diseases, translating into 18–30 million in the European Union and 263–446 million worldwide. The figures for uncommon respiratory disorders are also significant. Even with a reasonable estimate of 5% of rare diseases with a respiratory component, 1–2 million Europeans are likely to be affected by a rare respiratory disease. As a result, rare (respiratory) disorders are a public health and social issue [1,2,3].

The pathophysiology of rare diseases is unclear due to a lack of research, making the creation of safe and efficient medications, biologics, and medical technologies to prevent, diagnose, treat, or cure these diseases extremely difficult. All of this translates into significant challenges in obtaining public or private funding for rare disease research and assembling a sufficient number of patients for ensuring conclusive results from which health authorities could authorize the use of safe and effective pharmaceutical products to treat rare diseases. Collaboration is always required to increase the understanding and development of innovative medicines for these uncommon diseases. In recent years, health authorities worldwide have collaborated closely to solve these issues with the help of pharmaceutical corporations, scientists, and healthcare professionals. As a result of this partnership, new methods for improving the diagnosis, prognosis, and treatment of uncommon diseases have been established [3]. Patient organizations play a significant role in this regard [4]. The physical, emotional, and financial impacts of rare diseases on affected people and their families explain why those affected join groups dedicated to supporting research into the origins or causes of their illness and the development of effective medicines. A growing number of patient organizations promote and fund research projects, learn about and follow up on their findings, and partner with government agencies, the pharmaceutical sector, and clinical and academic researchers. To name a few, the American Association of patients with alpha-1 antitrypsin deficiency (AATD) has been funding research on this disease for years [5]. The Alpha-1 Spanish Association has recently announced the first edition of the Amadeu Monteiro Fellowships to encourage younger researchers to initiate new research projects on AATD [6].

As is the case with most rare diseases, patients with rare respiratory diseases face several problems, including underdiagnosis and delayed diagnosis, which, in many cases, have a detrimental influence on the prognosis of patients. In addition, the lack of prognostic biomarkers and disease-specific treatments are also challenges. These problems have been addressed in this special issue on rare respiratory diseases that cover, among others, conditions such as AATD, primary ciliary dyskinesia (PCD), pulmonary hypertension, and cystic fibrosis (CF).

Alpha-1 antitrypsin deficiency (AATD) is a potentially deadly hereditary type of chronic obstructive pulmonary disease (COPD). Precise diagnostic criteria are lacking and might take years to arrive, and late diagnosis is an almost impossible challenge. Over 3 million individuals worldwide have deficient allele combinations leading to AATD, with over 120,000 Europeans expected to have severe forms of the disease. Because the symptoms and signs are similar, AATD is easily confused with smoking-induced COPD or asthma. The average time between the onset of pulmonary symptoms and diagnosis is 8.3 years, and patients visit an average of 2.7 doctors before receiving a definitive diagnosis, which translates into more than 90% of AATD patients being undiagnosed. These figures are unacceptable because there is a specific treatment for the disease. Augmentation therapy, the only approved therapy to treat the disease, slows down the progression of the pulmonary disease and improves survival rates significantly [7].

Lack of awareness is one reason that accounts for underdiagnosis and delayed diagnosis. In this special issue, several strategies are described that may help to improve AATD diagnosis. Requena et al. showed significant gaps in knowledge about AATD and PCD among medical students and paediatricians. The authors suggest that all physicians responsible for detecting and diagnosing rare respiratory diseases should get additional training—allowing for early diagnosis, the implementation of preventive measures, and appropriate treatment in the early stages [8]. 

Expert centres on rare respiratory diseases that focus on both the experience and the number of patients will lead to a more effective approach in managing these patients, as described in the article by Lopez-Campos et al [9]. 

Annunziatta et al. studied rare variants in the geographic area of Naples (Italy). The authors’ findings may be useful for understanding the prevalence of AATD and its rare mutations, promoting early diagnosis and treatment for patients with chronic pulmonary disease and frequent exacerbations, and challenging the link between environmental causes of pulmonary damage, such as tobacco use [10].

Similar to AATD, the underdiagnosis of PCD is common. PCD symptoms are similar to those of other respiratory diseases [11]. To overcome these challenges, Armengot-Carceller et al. developed a diagnostic decision tree based on pansinusitis, situs inversus, periodicity, rhinorrhea, bronchiectasis, and chronic wet cough to classify new individuals. The authors concluded that the presence of all of these clinical symptoms in the same patient indicates a high risk of developing PCD [12]. However, validation of this diagnostic algorithm is still missing.

In addition, there is no gold standard for diagnosing PCD, and the currently available diagnostic methods are complex and not available in all hospital centres, leading to underdiagnosis in many cases. Several methods are currently being studied that will improve PCD diagnosis. Coles et al. have set up a new air–liquid culture procedure that produces high ciliation rates across three centers, minimizing patient recall for repeat brushing biopsies and improving diagnostic certainty. In addition, cryostorage of diagnostic samples was successful, facilitating PCD research [13]. Baz-Redon et al. demonstrated that an immunofluorescence-based method is a quick, low-cost, and reliable diagnostic test for PCD and is available in most hospitals. However, it cannot be used as an independent test [14].

Several authors have also studied the availability of prognostic biomarkers. Hernandez-Perez et al. demonstrated that the presence of a PI*Z allele seemed to be a risk factor for developing hepatic damage. Deficient AAT genotypes were linked to changes in liver enzymes, and low AAT levels were associated with high liver enzyme levels [15]. Pons et al. showed that abnormal liver enzymes are common in patients with AATD; however, most patients do not have significant liver fibrosis. The authors proposed using transient elastography to identify AATD patients with liver fibrosis, even if they have normal liver enzymes; this technique should be performed in all individuals with the Z allele to screen for liver disease [16].

In CF children, Stachowiak et al. discovered a profile of miRNAs, the expressions of which change during pulmonary exacerbations, and which are strongly linked to clinical outcomes [17].

The role of oxidative stress in the pathophysiology of rare respiratory diseases has also been discussed. In a review paper, Magallon et al. showed oxidative stress and increased biomarkers of oxidative damage in patients with AATD, idiopathic pulmonary fibrosis, and CF. As a result, targeting oxidative stress with antioxidant therapies is a rational approach to delaying disease progression and improving patient quality of life in all three conditions. In the case of PCD, the available data are limited, and further research is needed to determine the pathophysiological role of oxidative stress in the disease and, as a result, the possibility of administering antioxidant supplements [18]. A new method based on flow cytometry has been developed to investigate a comprehensive set of oxidative parameters in nasal epithelial cells, which might be useful in investigating respiratory diseases. This method has the benefit of using small samples and a non-invasive sampling technique [19].

Finally, the potential for new therapeutic strategies based on gene therapy has been explored, since no curative treatments are available for these diseases. As evidenced by the encouraging results in the preclinical and clinical phases, gene therapy is a promising alternative for the current therapies. However, further research is needed to ensure treatment safety and efficacy. In addition, new gene-editing tools used to correct mutations and enable cures for these diseases have been discussed [20].

In conclusion, this series of articles highlights some of the major problems that patients with rare diseases face and the need for further cooperative research between basic and clinical researchers to solve these problems.

## Data Availability

Not applicable.
